# Supplementary material for: Arabinosyltransferase C Mediates Multiple Drugs Intrinsic Resistance by Altering Cell Envelope Permeability in Mycobacterium abscessus
Source: Microbiol Spectr. 2022 Aug 10;10(4):e02763-21. doi: 10.1128/spectrum.02763-21 (PMC9430846; doi:10.1128/spectrum.02763-21)
Supplement: Supplemental file 1 — Supplemental material. Download spectrum.02763-21-s0001.pdf, PDF file, 0.5 MB [file spectrum.02763-21-s0001.pdf]

Supplementary Information

Arabinosyltransferase C Inhibitors may Enhance Activities of Multiple Drugs by Altering Cell Envelope Permeability in *Mycobacterium abscessus*

Shuai Wang[a,b,c,d,e], Xiaoyin Cai[b,c,d], Wei Yu[b,c,d,e], Sheng Zeng[b,d], Jingran Zhang[b,d,e,f], Lingmin Guo[b,c,d], Yamin Gao[b,c,d,e] Zhili Lu[b,c,d,e], H.M. Adnan Hameed[b,c,d,e], Cuiting Fang[b,c,d,e], Xirong Tian[b,c,d,e] Buhari Yusuf[b,c,d,e], Chiranjibi Chhotaray[b,c,d], M.D. Shah Alam[b,c,d,e], Buchang Zhang[g], Honghua Ge[g], Dmitry A. Maslov[h], Gregory M. Cook[i,j], Jiacong Peng[d,k], Yongping Lin[d,k], Nanshan Zhong[d,k,l] Guoliang Zhang[a*], Tianyu Zhang[b,c,d, e*]

[a] National Clinical Research Center for Infectious Diseases, Guangdong Provincial Clinical Research Center for Tuberculosis, Shenzhen Third People's Hospital，Shenzhen, 518055, China

[b] State Key Laboratory of Respiratory Disease, Guangzhou Institutes of Biomedicine and Health, Chinese Academy of Sciences, Guangzhou 510530, China

[c] University of Chinese Academy of Sciences, Beijing 100049, China

[d] Guangdong-Hong Kong-Macao Joint Laboratory of Respiratory Infectious Diseases, Guangzhou Institutes of Biomedicine and Health, Chinese Academy of Sciences, Guangzhou 510530, China

[e] China-New Zealand Joint Laboratory on Biomedicine and Health, Guangzhou, 510530, China

[f] School of Life Sciences, University of Science and Technology of China, Hefei, Anhui 230027, China

[g] Institutes of Physical Science and Information Technology, Anhui University, Hefei, 230601, China

[h] Laboratory of Bacterial Genetics, Vavilov Institute of General Genetics, Russian Academy of Sciences, Moscow, 119333, Russia

[i] Department of Microbiology and Immunology, School of Biomedical Sciences, University of Otago, Dunedin, New Zealand

[j] Maurice Wilkins Centre for Molecular Biodiscovery, The University of Auckland, Private Bag, Auckland, New Zealand

[k] State Key Laboratory of Respiratory Disease, National Clinical Research Center for Respiratory Disease, The National Center for Respiratory Medicine, The First Affiliated Hospital of Guangzhou Medical University, Guangzhou, 510230, China

[l] Guangzhou Laboratory, Bio-Island, Guangzhou, 510320, China

*Correspondence: Tianyu Zhang, zhang_tianyu@gibh.ac.cn; Guoliang Zhang, szdsyy@aliyun.com

Table S1 Information of transposon mutants hypersensitive to rifampicin

Mutant No.	Gene Name	Product
C9	*MAB_0122*	Hypothetical protein
C29	Between *MAB_0118c* and *MAB_0119c*	Probable superoxide dismutase<br>Hypothetical protein
F6	*MAB_1914*	Probable adenylate cyclase
E16	Between *MAB_1914c* and *MAB_1915*	Probable adenylate cyclase<br>Probable fatty-acid-coa ligase FadD
E21	*MAB_3044c*	tRNA delta(2)-isopentenylpyrophosphate transferase
F6	*MAB_1914*	Probable adenylate cyclase
I5	*MAB_3591c*	DNA-binding response regulator MtrA
R1	*MAB_3221c*	Conserved hypothetical protein
R16	*MAB_1915*	Probable fatty-acid-coa ligase FadD
Z6	*MAB_0189c*	Probable arabinosyltransferase C
Z7	*MAB_0189c*	Probable arabinosyltransferase C

Table S2 Primers used in this study

Primers	Nucleotide sequence (5’-3’)
**Tn-F**	CCCGAAAAGTGCCACCTAAATTGTAAGCG
**Z6-R**	CAACGCCAACGGCAGTCAA
**cz-0189cdown-F**	CTCGAGGTCGACGGTATCGATGAGGATATGGACGCACTCTTTCG
**cz-0189cdown-R**	GTGACGACCGAAGCTTTGCACAGCGGACTCTGGACAC
**cz-0189c-up-F**	TGCAAAGCTTCGGTCGTCACGGAGGAAGATG
**cz-0189c-up-R**	CGCTCTAGAACTAGTGGATCCCGCACCAAACACGCCACA
**Hinddifzeo-F***	CCCAAGCTTAACGCCGATAAGACACATTATGTCAGTTGAATTCCGA CCCGCACGAC
**Hinddifzeo-R***	CCCAAGCTTAACTGACATAATGTGTCTTATCGGCGTTTCAGTCCTGC TCCTCGGCCA
**0189cUD-R**	CGCACCAAACACGCCACA
**0189cUD-F**	GAGGATATGGACGCACTCTTTCG
**0189c-F(b)**	CCCTACCGTGCTGCTGATGC
**C0189c-R(c)**	ACCTTCCTTTGGGATGTCTTTGC
**JDZeoU(e)**	GGTGAATCCTCCTGAATATGTAGAG
**JDzeoD(f)**	CCGAGGAGCAGGACTGAAACGC
**JD0189cD(d)**	GGTTCGCATACCGCACATTG
**JD0189cU(a)**	CCGTGTATCTGTGGTCGTTGCT
**MtbembC-F**	GGCCAAGACAATTGCGGATCCCGGCGCACATAACAGCTACACCC
**MtbembC-R**	GTTAACTACGTCGACATCGATTCACCGGAAGCAGCGGAACG
**cz0189c-F**	GGCCAAGACAATTGCGGATCCCCCTACCGTGCTGCTGATGC
**cz0189c-R**	GTTAACTACGTCGACATCGATACCTTCCTTTGGGATGTCTTTGC

* The *dif* core sites are underlined.

Supplementary Figure 1:

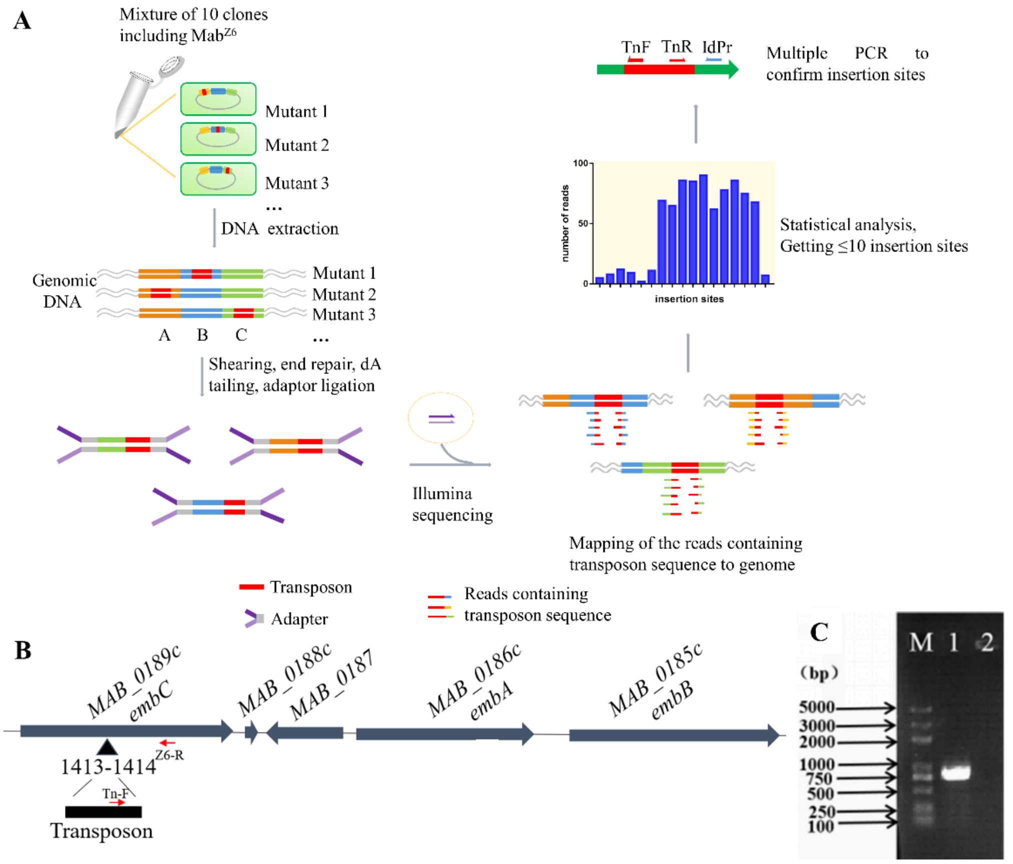

Fig. S1 Identification of transposon insertion sites. (A) Transposon insertion sequencing workflow. To identify the insertion sites more efficiently, 10 individual transposon insertion mutants were mixed for genomic DNA extraction. The sheared DNA fragments (containing ≤10 potential transposon insertion sites) were ligated to sequencing adaptors for subsequent high throughput sequencing. The reads containing transposon sequence were mapped to the *M. abscessus* genome, and only insertions with > 20 reads at specific site were considered to be potent insertions which were further identified in combination or individually by PCR. Lastly, the disrupted regions were confirmed by PCR using a pair of primers targeting Tn (TnF or TnR) and the disrupted gene (IdPr). (B) The *MAB_0189c* gene and adjacent genes are shown. Gray arrows represent open reading frames of genes and the black triangle indicates the insertion site of the Tn in the *MAB_0189c* gene. (C) PCR analysis of Tn insertion in *MAB_0189c* in Mab^{Z6} (lane 1) using the primers Tn-R and Z6-R (B). Mab^{Wt} (lane 2) was used as a control.

Supplementary Figure 2:

```
M. abscessus     VTTDEPLARRGDSTAGNATEKVAGNGDGEYRKARLLAIVTGFLGALLAIATPLLPVRQDTAQLNWPQNNTLASVDAPLIG     80
M. tuberculosis  MATEAAPPRIAVRLPSTSVRDAGAN....YRIARYVAVVAGLLGAVLAIATPLLPVNQTTAQLNWPQNGTFASVEAPLIG     76
M. smegmatis     ...............VTGPHAAGGS...NHRTARLVAIIAGLLGTLMAIATPLLPVEQTTAELNWPQNGVWQSVDAPLIG     62
Consensus                                      r ar  a   g lg   aiatpllpv q ta lnwpqn    sv aplig

M. abscessus     YVPTDLTITVPCAAAKGLDAHNN....VLLSTVPKQAPNAVDRGMLIQRSGGDLVVIVRNVPVVSAPFSEVLGPNCQRLE    156
M. tuberculosis  YVATDLNITVPCQAAAGLAGSQNTGKTVLLSTVPKQAPKAVDRGLLLQRANDDLVLVVRNVPLVTAPLSQVLGPTCQRLT    156
M. smegmatis     YVATDLTVTVPCQAAAGLVGPENRNRSVLLSTVPKQAPKAIDRGLLIERINNDLTVIVRNTPVVSAPLEQVLSPDCRYLT    142
Consensus        yv tdl  tvpc aa gl    n    vllstvpkqap a drg l  r   dl   vrn p v ap   vl p c  l

M. abscessus     VSAHSDKVTGKFVGLTQGPKDAKPGQPRAGERGGYDFRPQIVGIFTDLSGPAPAGLKLSATVDTRYSSSPTVAKLIAMIL    236
M. tuberculosis  FTAHADRVAAEFVGLVQGPNAEHPGAPLRGERSGYDFRPQIVGVFTDLAGPAPPGLSFSASVDTRYSSSPTPLKMAAMIL    236
M. smegmatis     FTAHADKVTGEFVGLTQGPDDDDPGEAVRGERSGYDFRPQIVGVFTDLSGPAPEGLQLSATIDTRYSTSPTLLKLLAMIV    222
Consensus          ah d v   fvgl qgp    pg    ger gydfrpqivg ftdl gpap gl  sa  dtrys spt  k  ami

M. abscessus     GVLLTAVSLVALHTLDTADGRRHKRFMPEGWWKPRPLDALVGAVLVWWHFVGANTSDDGYILTMARVAEGSGYMANYYRW    316
M. tuberculosis  GVALTGAALVALHILDTADGMRHRRFLPARWWSTGGLDTLVIAVLVWWHFVGANTSDDGYILTMARVSEHAGYMANYYRW    316
M. smegmatis     GVAMTVIALGALHVLDCADGRRHKRFLPSRWWSMTPLDGLVSAMLVWWHFVGANTADDGYILTMARVSEHAGYMANYYRW    302
Consensus        gv  t   l alh ld adg rh rf p  ww    ld lv a lvwwhfvgant ddgyiltmarv e  gymanyyrw

M. abscessus     FGTPESPFGWYYDLLTIWAHVSTASVWMRLPTLAMGLLCWAVISREVIPRLGHAARTNPVVPWTAAAMFLAFWLPFNNGL    396
M. tuberculosis  FGTPEAPFGWYYDLLALWAHVSTASIWMRLPTLAMALTCWWVISREVIPRLGHAVKTSRAAAWTAAGMFLAVWLPLDNGL    396
M. smegmatis     FGTPESPFGWYYDLLALWAHVSTASVWMRFPTLLMGLACWWVISREVIPRLGAAAKHSRAAAWTAAGLFLAFWLPLNNGL    382
Consensus        fgtpe pfgwyydll  wahvstas wmr ptl m l cw visreviprlg a        wtaa  fla wlp  ngl

M. abscessus     RPEPIIALGILLTWCSVERSIATNRLLPAAAACIIGALTLFSGPTGIASIGALLVAIGPLRTIVSKRAKRFGYAALLAPI    476
M. tuberculosis  RPEPIIALGILLTWCSVERAVATSRLLPVAIACIIGALTLFSGPTGIASIGALLVAIGPLRTILHRRSRRFGVLPLVAPI    476
M. smegmatis     RPEPIIALGILLTWCSVERGVATSRLLPVAVAIIIGALTLFSGPTGIAAVGALLVAIGPLKTIVAAHVSRFGYWALLAPI    462
Consensus        rpepiialgilltwcsver  at rllp a a iigaltlfsgptgia  gallvaigpl ti      rfg   l api

M. abscessus     LAAGLVTLIVIFRDQTLVGEIQANALKSAVGPSLNWFDEHVRYERLFLPTTDGAISRRFPVLALVIALGVAVAMTLRKNK    556
M. tuberculosis  LAAATVTAIPIFRDQTFAGEIQANLLKRAVGPSLKWFDEHIRYERLFMASPDGSIARRFAVLALVLALAVSVAMSLRKGR    556
M. smegmatis     AAAGTVTIFLIFRDQTLAAELQASSFKSAVGPSLAWFDEHIRYSRLFTTSPDGSVARRFAVLTLLLALAVSIAMTLRKGR    542
Consensus         aa  vt   ifrdqt   e qa   k avgpsl wfdeh ry rlf    dg   rrf vl l  al v  am lrk

M. abscessus     IPGTASGPSRRIIGITLISFVAIMFTPTKWTHHFGVFAGLAGSLGALAAVAVTAAAMRSPRNRTLYAAIVLFVLSLSFSS    636
M. tuberculosis  IPGTAAGPSRRIIGITIISFLAMMFTPTKWTHHFGVFAGLAGSLGALAAVAVTGAAMRSRRNRTVFAAVVVFVLALSFAS    636
M. smegmatis     IPGTALGPSRRIIGITIISFLAMMFTPTKWTHHFGVFAGLAGCLGALAAVAVTTTAMKSRRNRTVFGAAVLFVTALSFAT    622
Consensus        ipgta gpsrriigit isf a mftptkwthhfgvfaglag lgalaavavt  am s rnrt   a v fv  lsf

M. abscessus     VNGWWYVSNFGVPWSNSFPQWHFGISTVFFGLSVLAILIAAWMHFTGRDHPGRTP....VHPVLARLAESPLAVGTWIVV    712
M. tuberculosis  VNGWWYVSNFGVPWSNSFPKWRWSLTTALLELTVLVLLLAAWFHFVANGDGRRTARPTRFRARLAGIVQSPLAIATWLLV    716
M. smegmatis     VNGWWYVSNFGVPWSNSFPEFKFGFTTMLLGLSVLALLVAAWFHFSGRDVSPDRP.....QRRWQRLLVAPLAVATWALV    697
Consensus        vngwwyvsnfgvpwsnsfp       t    l vl  l aaw hf                         pla  tw  v

M. abscessus     VWSIFSLTAGMINQYPAWSVGRSNLDALRGNGCGLANDVMVEEDPNAGMLQPIDAPIGQALAADTNIMFDPNGIPSDVSA    792
M. tuberculosis  LFEVVSLTQAMISQYPAWSVGRSNLQALAGKTCGLAEDVLVELDPNAGMLAPVTAPLADALGAGLSEAFTPNGIPADVTA    796
M. smegmatis     IFEVVSLTLGMINQYPAWSVGRSNLNALTGKTCGLANDVLVEQNANAGMLTPIGEPAGQALGAVTSLGFGPNGIPSDVSA    777
Consensus             slt  mi qypawsvgrsnl al g  cgla dv ve   nagml p   p   al a     f pngip dv a

M. abscessus     DEENNPQGSDSFVERDKSGNANGSQ.DTEGGTTIAAGVNGSRARLPFDLKPETTPVMGSYQVGPQRSARLLSSWYRLPPK    871
M. tuberculosis  DPVMERPGDRSFLNDD..GLITGSEPGTEGGTTAAPGINGSRARLPYNLDPARTPVLGSWRAGVQVPAMLRSGWYRLPTN    874
M. smegmatis     DPVMEQPGTDNFADSD.SGVVTGTEVGTEGGTTAAAGINGSRARLPYGLNPATTPVLGSWRSGTQQPAVLRSAWYRLPDR    856
Consensus        d      g   f   d  g   g    teggtt a g ngsrarlp  l p  tpv gs   g q  a l s wyrlp

M. abscessus     D..ATKPLLVLAAAGRFDPVELQVQFAGED....GKVLGAISFADLGPSPAWRNLRMSRDAIPTQATRIRLLVTDDDLAP    945
M. tuberculosis  EQRDRAPLLVVTAAGRFDSREVRLQWATDEQAAAGHHGGSMEFADVGAAPAWRNLRAPLSAIPSTATQVRLVADDQDLAP    954
M. smegmatis     D..QAGPLLVVSAAGRFDQGEVEVQWATDEQAAANEPGGSITFGDVGAAPAWRNLRAPLSSIPPEATQIRLVASDDDLAP    934
Consensus              pllv  aagrfd  e   q a           g   f d g  pawrnlr     ip  at  rl   d dlap

M. abscessus     QHWVAITPPRVPSLRSLQAVVGS.DPVFLDWLVGLAFPCQRPFGHKNGVIEIPKWRILPDRFGAEANSPVMDYLGGGPLG   1024
M. tuberculosis  QHWIALTPPRIPRVRTLQNVVGAADPVFLDWLVGLAFPCQRPFGHQYGVDETPKWRILPDRFGAEANSPVMDHNGGGPLG   1034
M. smegmatis     QHWIALTPPRIPELRTLQEVVGSSDPVMLDWLVGLAFPCQRPFDHRYGVVEVPKWRILPDRFGAEANSPVMDYLGGGPLG   1014
Consensus        qhw a tppr p  r lq vvg  dpv ldwlvglafpcqrpf h  gv e pkwrilpdrfgaeanspvmd  gggplg

M. abscessus     ITELLLKATPVPTYLQNDWFRDWGALQRFTPYYRNATEAQLQLGTAVHSGLWTPGPLRKS                       1084
M. tuberculosis  ITELLMRATTVASYLKDDWFRDWGALQRLTPYYPDAQPADLNLGTVTRSGLWSPAPLRRG                       1094
M. smegmatis     ITELLLRPSSVPTYLKDDWYRDWGSLQRLTPWYPDAQPARLDLGTATRSGWWSPAPLRLS                       1074
Consensus        itell     v  yl  dw rdwg lqr tp y  a  a l lgt   sg w p plr
```

Fig. S2 The alignment of amino acid sequences of EmbC proteins from *M. abscessus* (MAB_0189), *M. tuberculosis* and *M. smegmatis*. MAB_0189 showed 68.40% identity with EmbC of *M. tuberculosis* and 68.94% identity with EmbC of *M. smegmatis*.

Supplementary Figure 3:

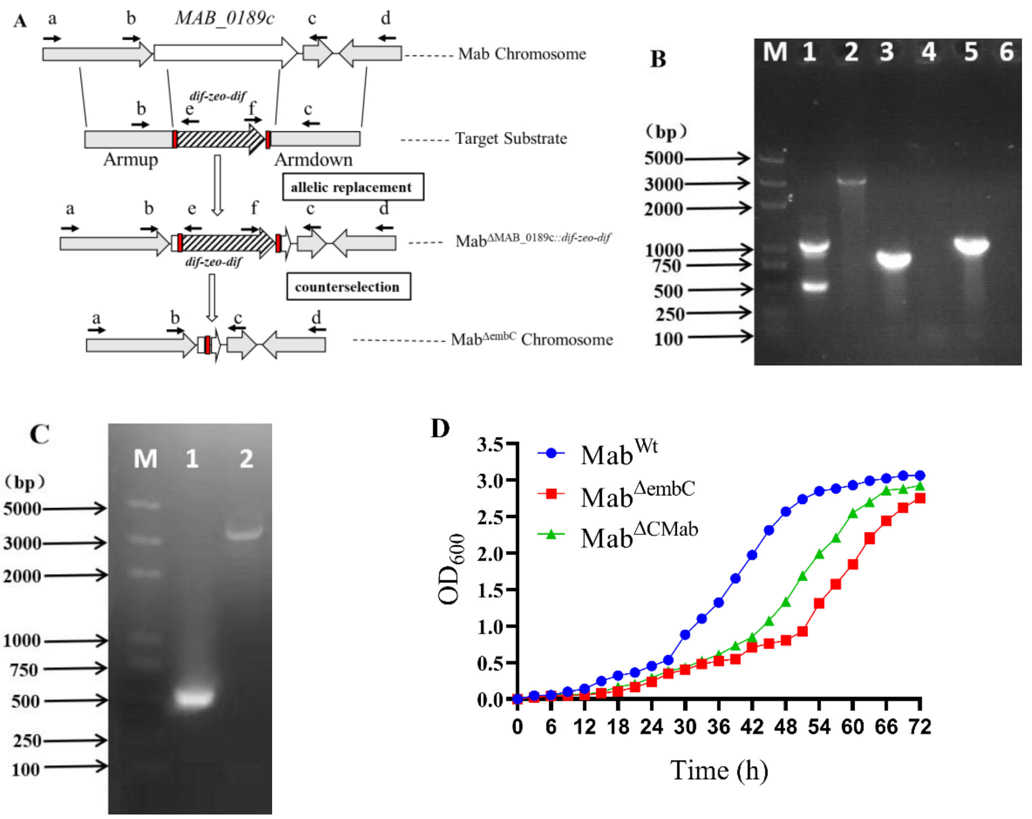

Fig. S3 Selectable marker-free deletion of *MAB_0189c*. (A) Diagrammatic illustration of the construction of a selectable marker-free *MAB_0189c* deletion in *M. abscessus*. (B) Lane M, DNA marker; lane 1, PCR products from *M. abscessus* ΔMAB_0189c::*dif-zeo-dif* using primers b+c; lane 2, PCR products from Mab^{Wt} using primers b+c; The pattern is consistent with the predicted wild-type fragment at 3.7 kb, knock out fragments at 1.1 kb and 0.5 kb. lane 3, PCR products from *M. abscessus* ΔMAB_0189c::*dif-zeo-dif* using primers a+e; lane 4, PCR products from Mab^{Wt} using primers a+e; The pattern is consistent with the predicted knock out fragment at 0.9 kb, wild-type no fragments. lane 5, PCR products from *M. abscessus* ΔMAB_0189c::*dif-zeo-dif* using primers f+d; lane6, PCR products from Mab^{Wt} using primers f+d. The pattern is consistent with the predicted knock out fragment at 1.1 kb, wild-type no fragments. (C) Lane M, DNA marker; lane 1, PCR products from $Mab^{\Delta embC}$ using primers b+c; lane 2, PCR products from Mab^{Wt} using primers b+c. The pattern is consistent with the predicted wild-type fragment at 3.7 kb, knock out fragments at 0.5 kb. (D) Growth analysis of the Mab^{Wt}, $Mab^{\Delta embC}$ and $Mab^{\Delta CMab}$ strains in 7H9 broth at 37°C. The growth rate of $Mab^{\Delta embC}$ was slower than Mab^{Wt}.

Supplementary Figure 4:

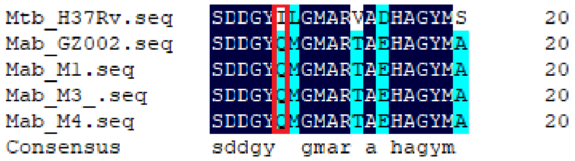

Fig. S4 Alignment of resistance-determining region of the EmbB proteins of different *M. abscessus* isolates and *M. tuberculosis* H37Rv. The red box indicates that isoleucine at position 281 of EmbB is replaced by glutamine in *M. abscessus*.
